# Advancing Global Harmonization: Implementing Global Dose Form Attributes for Medicinal Products Identification

**DOI:** 10.1007/s43441-025-00838-3

**Published:** 2025-08-07

**Authors:** Emelie Ahnfelt, Olof Lagerlund, Jenny Klint, Malin Fladvad, Christopher Jarvis, Ta-Jen Chen, Panagiotis Telonis, Ronald Fitzmartin

**Affiliations:** 1 Uppsala Monitoring Centre, Uppsala, Sweden; 2 European Directorate for the Quality of Medicines and HealthCare, Strasbourg, France; 3 United States Food and Drug Administration, Silver Spring, USA; 4 European Medicines Agency, Amsterdam, Netherlands; 5 Decision Analytics LLC, Florida, United States

**Keywords:** Standardize dose forms, Dose form terminologies, Global Medicinal Harmonization, International Organization for Standardization (ISO), Identification of Medicinal Products (IDMP), Pharmacovigilance

## Abstract

The global pharmaceutical market includes a wide range of medicinal products, which makes it difficult to achieve consistent identification and classification across different regions. To address this, the International Organization for Standardization (ISO) developed five standards that together create a framework for the Identification of Medicinal Products (IDMP). Implementing the ISO IDMP standards globally necessitates collaboration to ensure consistent data. As part of these efforts, investigations were conducted to explore the feasibility of introducing global dose form attributes, which aim to standardize dose forms worldwide. These characteristics, defined by the European Directorate for the Quality of Medicines (EDQM), include the administrable basic dose form, administration method, intended site, and release characteristics. The global dose form attributes were successfully applied to data from nine countries, representing six dose form terminologies or lists, and were successfully assigned to over 99% of medicinal products. The process of assigning global dose form attributes was improved by mapping local dose form terminologies to global dose form attributes. The results from these studies were used to develop business rules for assigning the global dose form attributes.

## Harmonizing Medicinal Products: A Global Perspective

The global pharmaceutical market consists of an extensive array of medicinal products, ranging from painkillers, antibiotics, and vaccines to antineoplastics and more advanced products such as cell and gene therapies. The same products were often described similar between countries or regions, but not consistent. To address this diversity issue, the International Council for Harmonisation of Technical Requirements for Pharmaceuticals for Human Use (ICH) endorsed a concept paper, in mid-2000 on the topic of standardization of medicinal products. This paper laid the foundation of the work carried out by the International Organization for Standardization (ISO), culminating in the publication in 2012 of the comprehensive suite of standards, collectively known as the Identification of Medicinal Products (IDMP) framework [1, 2]. Although the development towards global standardization has started, there is, for example, no globally unified dose form terminology. Medicinal products’ dose forms are often based on national or regional terminologies or lists maintained by regulatory authorities. This diversity in terminology poses a significant challenge when it comes to consistent identification and classification of medicinal products across different regions.

Successful global implementation of the ISO IDMP standards requires harmonization and consistency in the data generated across different countries [3–7]. To facilitate this, the Global IDMP Working Group (GIDWG) was established. The founding members of GIDWG include the European Medicines Agency (EMA), the US Food and Drug Administration (US FDA), and Uppsala Monitoring Centre (UMC) [8–10]. The group's primary objective is to implement the IDMP standards to ensure consistent global identification of medicinal products, particularly through the Pharmaceutical Product Identifier (PhPID) described in ISO IDMP standard 11616.

The PhPID can play a critical role in various applications such as pharmacovigilance, managing drug shortages, and enabling cross-border prescriptions. It facilitates the aggregation of pharmacovigilance data for signal detection and aids in finding comparable products, whether for cross-border prescriptions or addressing drug shortages within a specific region or country.

**Figure 1:**
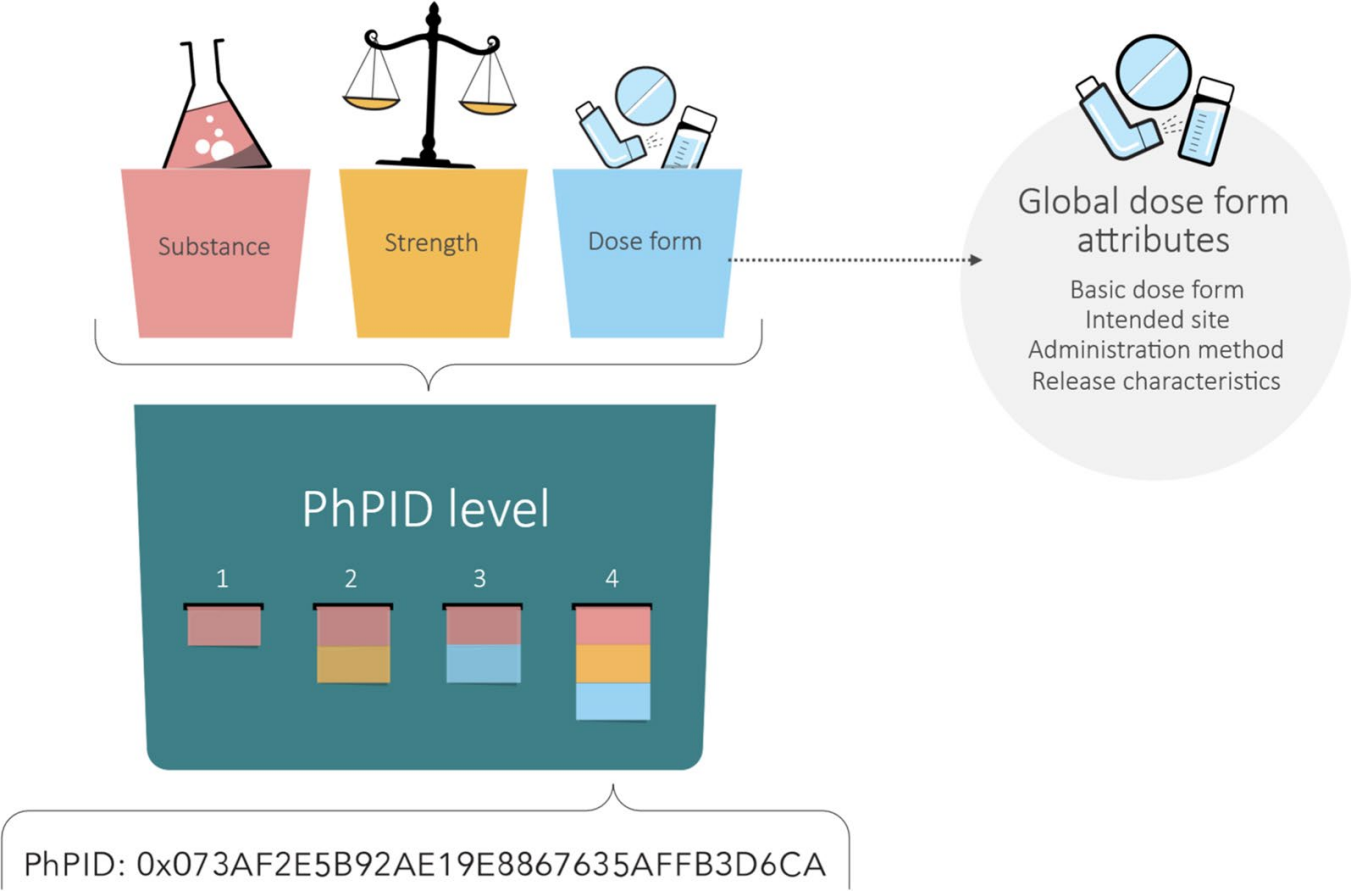
The four levels of Pharmaceutical Product Identifier (PhPID).

The PhPID is generated based on information about the substance [11, 12], administrable dose form [13], and strength [14], all defined in the ISO IDMP standards. Each PhPID is generated on four levels: Level 1 identifies the active substance(s), which form the basis for subsequent levels; Level 2 includes the active substance(s) and strength; Level 3 includes the active substance(s) and dose form; and Level 4 combines active substance(s), strength, and dose form (Fig. 1). This article focuses on the global harmonization of dose forms for the creation of global PhPIDs. The study was conducted in three parts between 2019 and 2024, consisting of an initial investigation of a dose form mapping exercise (Part I) and two medicinal product validation studies (Part II and III) where the complexity of the medicinal products validated were increased in Part III with regards to substances as well as dose forms.

## Part I: Dose Form Mapping Exercise

Medicinal products’ dose forms are often based on national or regional lists or terminologies maintained by regulatory authorities, which are typically restricted to regional or local jurisdictions. These terminologies vary in granularity and semantics, leading to inconsistencies and a lack of uniformity across regions. A key challenge in achieving global harmonization is the ability to use different dose form terminologies across regions while still enabling seamless global data exchange.

To explore this challenge, a mapping exercise (Part I) was conducted in 2019 to assess whether existing pharmaceutical dose form terminologies or dose form lists could be aligned for global use. The exercise involved evaluating one-to-one mappings between the European Directorate for the Quality of Medicines & HealthCare (EDQM) pharmaceutical dose forms [15] and those used by the U.S. FDA, Health Canada, Systematized Nomenclature of Medicine (SNOMED) [16], and the Clinical Data Interchange Standards Consortium (CDISC) [17]. The EDQM Standard Terms were chosen due to its adherence to ISO 11239 with a highly granular hierarchy describing dose form attributes which enables information exchange independent of regional terminological variations.

**Figure 2:**
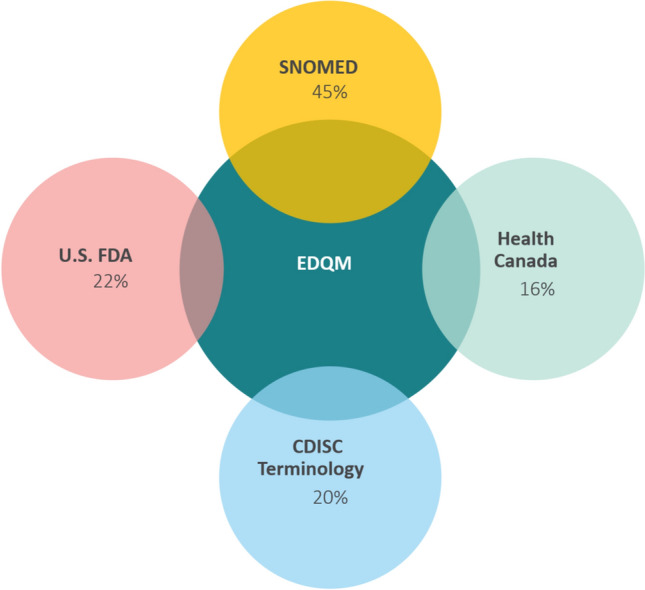
Regions use their own set of terminologies for dose form, with varying levels of granularity. This variation makes mapping between terminologies challenging. The one-to-one mapping (%) of dose form terminologies or dose form lists of the U.S. FDA, Health Canada, SNOMED, and CDISC to the EDQM pharmaceutical dose form, performed in 2019, is depicted above.

The results showed varying degrees of alignment (Fig. 2). One-to-one matches between the EDQM pharmaceutical dose forms and dose forms used by Health Canada and the US FDA were 16% and 22%, respectively, while CDISC had a 20% match, and SNOMED had a 45% match. These findings highlight the challenges posed by differing levels of granularity in dose form terminologies or dose form lists, complicating efforts to harmonize them on a global scale.

## The EDQM Dose Form Terminology

**Figure 3:**
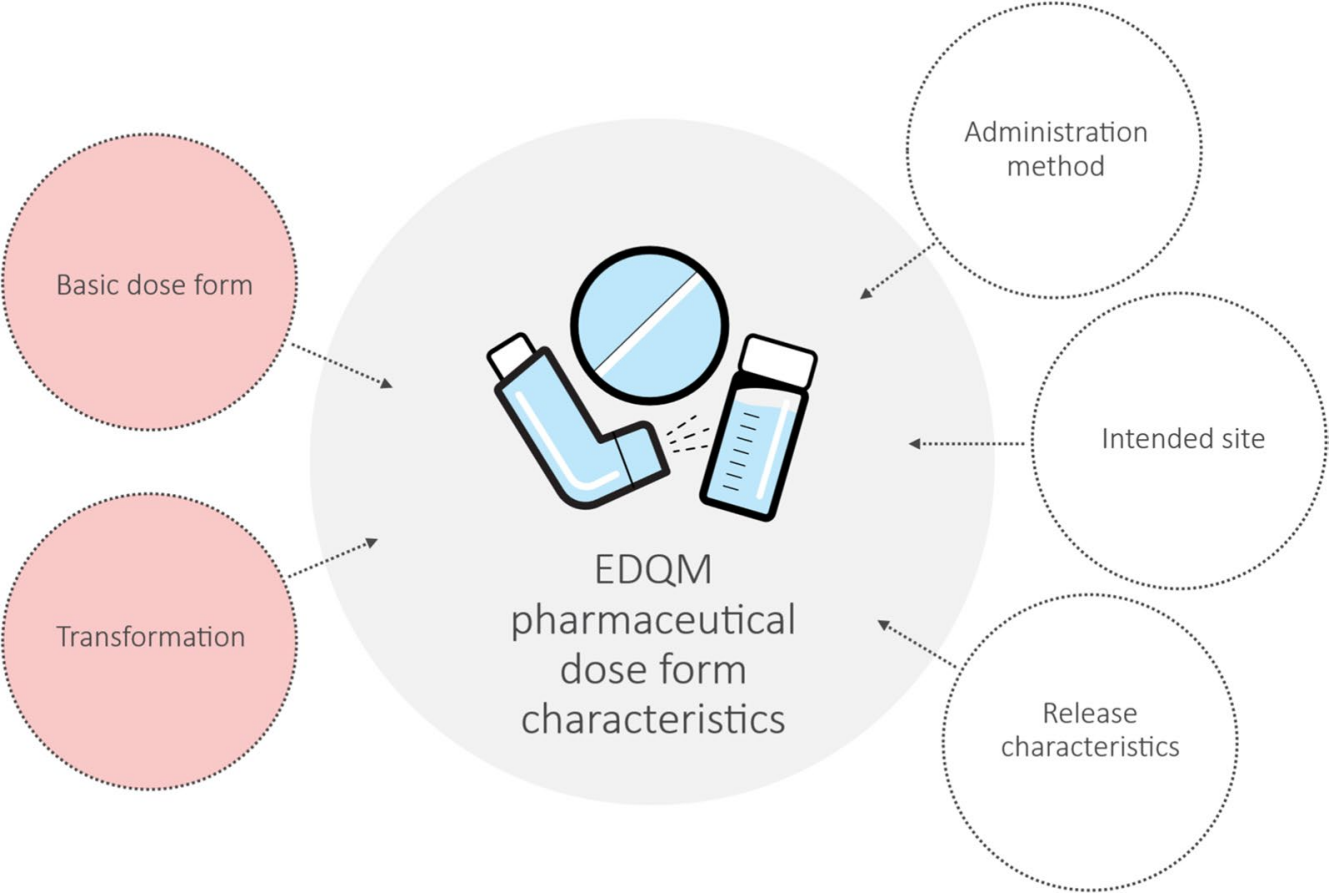
The five components defined by EDQM: —basic dose form, transformation, intended site, administration method, and release characteristics—used to describe the EDQM pharmaceutical dose form.

Since one-to-one matches between the pharmaceutical dose form terminologies and the investigated dose form terminologies were less than 45%, a proposal was made by US-FDA to use a centrally maintained set of dose form attributes to facilitate the global harmonization of regional and local dose form terminologies. EDQM [15] has established a comprehensive terminology, known as Standard Terms, which includes dose forms, dose form attributes and adheres to the ISO 11239 standard [13]. These Standard Terms serve as a robust and hierarchical reference system for assigning pharmaceutical dose forms. The EDQM dose form terminology comprises five essential components: basic dose form, transformation, release characteristics, intended site, and administration method (see Fig. 3).

In the EDQM Standard Terms, the pharmaceutical dose form is critical for describing both the manufactured item (as it appears in packaging by the manufacturer) and the pharmaceutical product (intended for patient administration after any necessary transformations) [15]. The manufactured item is referred to as the “Manufactured Dose Form”, while the product ready for administration is called the “Administrable Dose Form”.

Basic dose form is a general representation of the pharmaceutical dose form. Transformation describes if and how the manufactured item is transformed before administration to the patient. Intended site describes where the product is administered, while administration method describes how the product is administered. Finally, release characteristics describe when the product is made available in the body. Together, these five components collectively describe the EDQM pharmaceutical dose form.

Each pharmaceutical dose form within the EDQM terminology is systematically organized by its state of matter, followed by its basic dose form. The basic dose form is a generalized category that includes various pharmaceutical formulations, such as tablets, capsules, creams, ointments, solutions, and emulsions. The basic dose form serves as a valuable tool for defining and harmonizing dose form information associated with these products. Furthermore, the basic dose form acts as a generalized version of specific pharmaceutical dose forms, facilitating the grouping of related forms according to the structure outlined by EDQM Standard terms.

To further the goal of global harmonization of medicinal products, the use of EDQM terminology to consistently describe medicinal products worldwide was explored, particularly within the context of global pharmaceutical product identification. The aim was to investigate tools and processes to achieve global harmonization.

## Part II: Introduction of Global Dose Form Attributes

**Figure 4:**
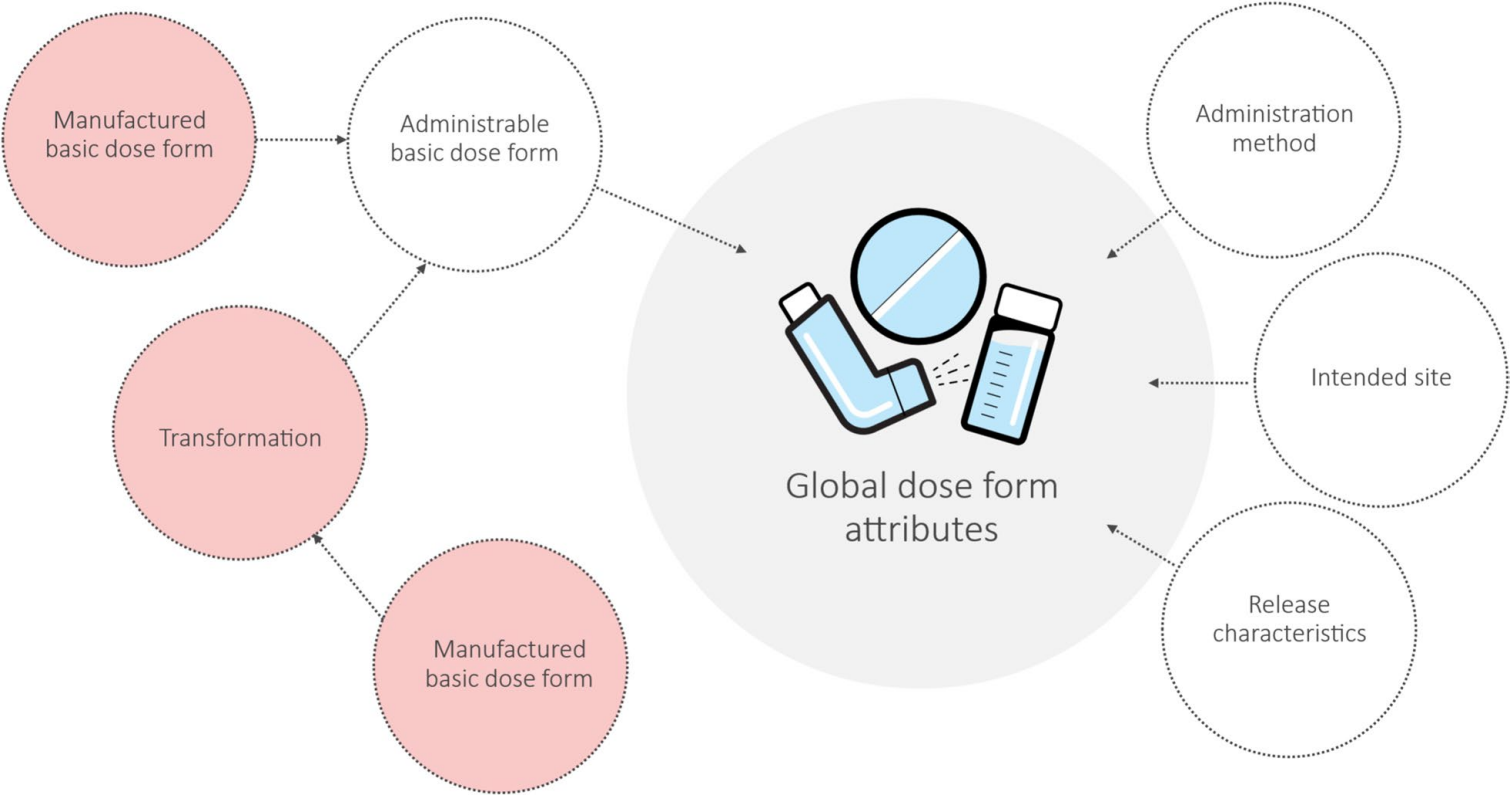
During the dose form study, four dose form attributes were used to increase the possibility of global harmonization of dose form terminologies. The attribute “transformation” from the EDQM dose form terminology is not used, as only administrable dose forms are included in the global dose form attributes. For pharmaceutical dose forms that must undergo a transformation before being administered to the patient, the manufactured basic dose form is replaced with the administered basic dose form.

The EDQM Standard Terms and their corresponding definitions were chosen as the basis for this centrally maintained set of standard terms. Four attributes—basic dose form, administration method, intended site, and release characteristics—were chosen to serve as descriptors (see Fig. 4). These are referred to as the global dose form attributes in this document.

According to ISO 11616 [2], the dose form should be the form that is administered to the patient. To adhere to this, the administered dose form is used, and it also replaces the manufactured dose form when a transformation was needed before administration (Fig. 4).

As only administrable dose forms are used in PhPID, the attribute for transformation was excluded from the global dose form attributes.

For example:

1. For a cream, there is no transformation and therefore the basic dose form for both the manufactured and administrable product is the same, namely cream.
2. For a powder for solution for injection, the manufactured basic dose form is powder, but the administrable basic dose form is a solution, which is created after the powder is dissolved. The basic dose form used in PhPID will be solution.

Part II of the study investigated if introducing the global dose form attribute terms could enable consistent data generation within and between organizations. To do this, 37 substances, originating from the up-scaling of the global univocal identification of medicines (UNICOM) pilot list [18], were chosen, since it was a set selected to present challenges in medicinal product identification [19] of substances. Medicinal products marketed in the US were collected, and each medicinal product was assigned a set of global dose form attributes. This assignment was done by UMC in collaboration with the US FDA. To assess whether global dose form attributes could be used for harmonization between regional and national dose form terminologies, the scope was expanded to include four additional countries and their corresponding dose form terminologies. The 37 substances were used to collect medicinal products from Belgium, Brazil, Finland, and Norway. The dose forms were validated using the information in the corresponding Summary of Product Characteristics (SmPC) or label, followed by assigning the global dose form attributes.

The study resulted in the assignment of global dose form attributes for 5,600 medicinal products. During the study, discrepancies were identified in how individuals and organizations assigned the global dose form attributes. Additionally, vague or incomplete information in SmPCs and labels created significant challenges during validation. Some medicinal products were described using multiple values for the global dose form attribute terms, indicating a need for a clear process on how to select the appropriate values. Finally, there was a need to investigate whether the global dose form attributes could be applied to additional dose form terminologies and whether the process of assigning the attributes to medicinal products was scalable.

## Part III: Implementation and Scalability of Global Dose Form Attributes

The study was further extended, in the third part, with the goal of implementing and scaling the attributes globally. It investigated whether the global dose form attributes could be applied to a broader range of diverse substances and dose forms beyond those initially studied i.e. more focus on variety than quantity regarding medicinal products. The study also examined medicinal products with multiple or ambiguous terms and assessed the scalability of included dose forms and efficiency of the validation process for assigning these attributes.

The collection of medicinal product data was expanded, with approximately 350 substances selected, allowing for an increased number of dose forms to be evaluated. To limit the number of medicinal products, 3,200 were selected—comprising three products per substance and dose form—from eight countries: Brazil, Canada, the European Union (represented by Croatia, France, and Greece), Norway, Switzerland, and the US. The medicinal product data was validated by matching the global dose form attributes to the information provided in each product's SmPC or label. The global scope was further broadened by investigating products on the Japanese market with a subset of the substances. Of the 350 substances investigated seventeen substances were selected. Using the same approach as for the other countries, selecting three medicinal products for each substance and dose form, a total of 148 corresponding Japanese medicinal products were validated. To retrieve comparable results during the study, changes in the Standard Terms [15] due to updates from EDQM were not implemented.

**Figure 5:**
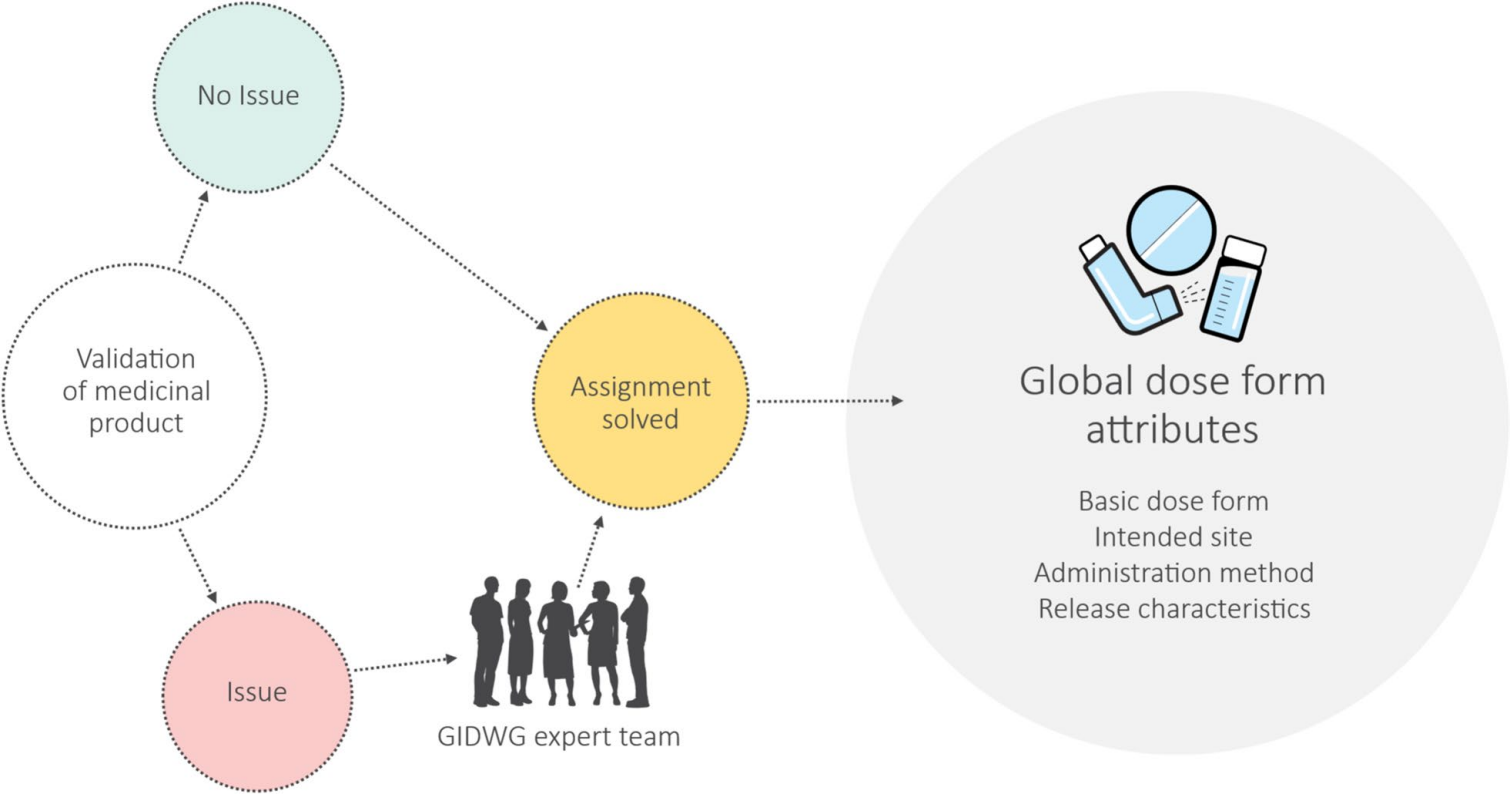
Validation process for selecting global dose form attributes for medicinal products.

The validation process was further refined during this study. Findings and issues were continuously collected, summarized, categorized, and escalated internally or referred to the GIDWG expert group [20] for guidance and decision-making (Fig. 5). The decisions were used to refine and formalize the GIDWG business rules, which formed the basis for assigning global dose form attributes. For example, the business rules specify the allowed number of terms for each attribute—one term for basic dose form and release characteristic, and multiple terms for intended site and administration method.

Throughout the study, over 99% of the 3,200 medicinal products were successfully assigned all four global dose form attributes. The 1% that could not be assigned the global dose form attributes were due to a lack of information in the SmPC. Sixty-three unique combination sets of global dose form attributes were assigned to one or more medicinal products. Of these 63 sets, 47 corresponded to a single EDQM pharmaceutical dose form, indicating the same level of granularity for the two systems. For example, the EDQM term “eye drops, emulsion” was assigned the global dose form attributes “emulsion, instillation, ocular, conventional”. Another example was the term “prolonged-release solution for injection” which was assigned the attributes “solution, injection, parenteral, prolonged”. For the Japanese subset it was possible to assign global dose form attributes to all included medicinal products. This resulted in fifteen sets of global dose form attributes of which ten corresponded to a single EDQM pharmaceutical dose form.

Some of the EDQM pharmaceutical dose forms include more granular information than the corresponding dose form attributes. For example, the EDQM terms “capsule, hard” and “capsule, soft” were aggregated to the same set of global dose form attributes: capsule, swallowing, oral, conventional. Another example was the four terms “coated tablet, film-coated tablet, tablet, tablet with sensor” which all were aggregated to the attributes “tablet, swallowing, oral, conventional”. During the study, 16 unique combinations of global dose form attributes corresponded to two or more EDQM pharmaceutical dose forms. For the Japanese subset five combinations of global dose form attributes corresponded to two or more EDQM pharmaceutical dose forms. All 15 sets of global dose form attributes in the Japanese subset overlapped with the 63 unique global attributes sets.

Five sets of global dose form attributes assigned to medicinal products did not correspond to any EDQM pharmaceutical dose form, demonstrating the flexibility of the global dose form attributes. One example was the U.S. local dose form “tablet, orally disintegrating, delayed release,” which was assigned the global dose form attributes “tablet, orodispersion, oral, delayed” which did not correspond to an EDQM pharmaceutical dose form. For the Japanese data, all global dose form attributes corresponded to an EDQM pharmaceutical dose form.

There were challenges when some SmPCs and labels lacked necessary information for accurate assignment of the global dose form attributes. For example, the manufactured dose form effervescent tablets sometimes lacked information on which administrable dose form it corresponded to—”solution” or “suspension”. This issue was addressed by a business rule assigning the term “solution” in such cases.

To improve global aggregation, rules for harmonization of global dose form attributes were implemented (see Table 1). For example, only the term “injection” was used for the administration method, excluding “infusion”. In another example, the term “syrup” was not used; instead, the appropriate basic dose form “solution” or “suspension” was selected, which required manual validation of each SmPC or label.

**Table 1:** Harmonization examples of global dose form attributes.

Description in SmPC or label	EDQM global dose form characteristic	Possible EDQM term	Harmonized global dose form attributes
Plaster or patch	Basic dose form	Plaster and patch	Patch
Syrup	Basic dose form	Solution or suspension	Solution or suspension, depending on the description in the corresponding SmPC or label
Dispersion or suspension	Basic dose form	Dispersion or suspension	Suspension
Injection and/or infusion	Administration method	Injection or infusion	Injection
Tablets that are: chewed or dissolved in the mouth; chewed or swallowed whole; chewable or dispersible	Administration method	Chewing and swallowing	Chewing and swallowing
Cutaneous drops	Administration method	Application and/or instillation	Application*
Oral drops	Administration method	Instillation and/or swallowing	Instillation and swallowing
Topical preparations with multiple intended sites, e.g. cutaneous and more	Intended site	Cutaneous, rectal, oromucosal, urethral	Cutaneous/transdermal for multiple intended sites.
Topical preparations with one intended site, e.g. rectal	Intended site	Rectal	Rectal
Eye/ear drops	Intended site	Ocular and auricular	Ocular and auricular
Patches	Release characteristic	Conventional and/or prolonged	Local effect conventional, systemic effect prolonged

^*^Instillation should not be assigned even though the solution is dropped to the skin.

For intended site, several terms were often described in the SmPC or label. The following business rule was applied for harmonization purposes: if a medicinal product could be used in more than one way, the intended site was assigned based on the primary use, or on the term with the strictest microbiological requirements. For example, a solution for injection that could also be administered by a nasogastric tube was assigned the intended site “parenteral” as the primary use due to the strictest microbiological requirements. Consequently, the intended site “gastric” was not assigned. In cases where it was not possible to identify a primary use or determine the strictest microbiological requirements, several characteristics were used to indicate two or more uses.

During the process of selecting release characteristics, it was observed that this information could be found in different sections of the SmPC or label, such as the product name, dose form name, or the pharmacokinetics sections. It was also noted that terms were used inconsistently, and this led to an increased amount of manual work, required to identify and assign a correct release characteristic term to the medicinal products.

For example, when harmonizing the release characteristics of patches, the term “conventional” was assigned for local effects, while “prolonged” was used for systemic effects (Table 2). If no information about release characteristics was found, the term “conventional” was assigned. Additionally, the EDQM terminology suggests using the term “modified release” specifically for pulsatile release. However, it was observed that this term was used to describe prolonged or delayed release characteristics for some medicinal products.

**Table 2:** Harmonization for multiple or combinations of release characteristic terms.

Release characteristics in SmPC/label	Combinations of terms	Harmonized release characteristics
Conventional and prolonged	Combination of conventional release and prolonged release	Prolonged
Delayed and prolonged	Combination of delayed release and prolonged release	Prolonged
Conventional and delayed	Combination of conventional release and delayed release with at least one ingredient exhibiting both release characteristics	Prolonged
	Combination of conventional release and delayed release where no ingredient exhibits both release characteristics	Delayed

Defining the release characteristics for certain products also posed challenges. To address this, the EDQM definition was complemented with detailed clarifications in the GIDWG Business Rules [20], providing guidance for assigning release characteristics. The important element to consider was when the drug becomes available in the body, meaning the time from injection/ingestion/application to the pharmacological response of the drug as described in the SmPC. This does not specifically refer to the time of release but may also consider delayed/prolonged/modified absorption. Often, a comparison was made to a conventional release product with the same active ingredient(s).

Another finding was that products with combinations of release characteristics were described differently in different regions. To address this, it was decided to harmonize these medicinal products according to the USP nomenclature guidelines [21] (Table 2).

## Part III: Mapping of Local Dose Forms to Global Dose Form Attributes

Part III of the study also aimed to improve the efficiency of assigning global dose form attributes. For countries using EDQM pharmaceutical dose forms, assigning the global dose form attributes were straightforward since each EDQM pharmaceutical dose form corresponded to a set of global dose form attributes. However, adjustments were necessary to ensure compliance with the business rules, such as using the administrable dose form instead of the manufactured dose form.

To improve the efficiency of medicinal product validation knowledge-based rules were applied for mapping other regional and local dose form terminologies to global dose form attributes. Furthermore, it was observed that additional information about the route could assist in assigning the global dose form attributes. During the validation of each medicinal product, manual corrections of the attributes were always possible.

A total of 188 local dose forms were evaluated. For nine local dose forms, no assignment of the attributes was possible. For the remaining 179 local or regional dose forms, one or more of the global dose form attributes could be assigned. For 42 of these 179 dose forms, all four global dose form attributes were assigned. During the validation of each medicinal product, the attributes that could not initially be assigned in the mapping process were identified based on the product SmPC.

## Discussion

Part I-III of the study highlighted the usefulness and robustness of the EDQM dose form terminology, which has previously been demonstrated [22, 23]. During the implementation of the global dose form attributes (Part II-III) as a common denominator for describing local dose forms in a global setting, a large diversity of dose forms was investigated, covering the most used EDQM pharmaceutical dose forms. All four global dose forms attributes were successfully assigned to more than 99% of the 3,348 medicinal products in Part III of the study.

The primary reason for not achieving complete assignment of the global dose form attributes was the lack of information in the SmPC or label. For example, some medicinal products with the dose form “syrup” lacked information about whether they corresponded to a suspension or solution. Other instances of missing information included a coated pill with an unclear description of the administration method and a gel cream where it was unclear whether the basic dose form should be classified as gel or cream. To resolve these issues, the missing information needs to be collected from other sources, or new business rules need to be formalized, to enable the assignment of global dose form attributes.

As shown in this study, data aggregation by harmonising dose forms could aid in mapping between dose form terminologies, since not all terminologies have the same level of granularity. It was also suggested that the aggregation of local dose forms to global dose form attributes could improve signal detection in pharmacovigilance by facilitating the collection of signals. Examples include aggregating hard and soft capsules under the unified term “capsule”.

On the other hand, some harmonisation of data should be implemented with thoughtful assessment. For example, the aggregation of the EDQM pharmaceutical dose forms—coated tablet, film-coated tablet, tablet, and tablet with sensor—to the global dose form attributes “tablet, swallowing, oral, and conventional”. Here, the “tablet with sensor” highlighted a limitation of the global dose form attributes, since it is not comparable to the other pharmaceutical dose forms. Another clinically relevant example was harmonising the administration method for parenteral medicinal products where the terms “infusion” and “injection” were harmonised to a single term “injection”. While being necessary for global harmonisation it shows a potential limitation with harmonisation and data aggregation.

Another instance of data harmonisation was the exclusion of the intended site term “gastric” following the rule of only assigning the intended site with the strictest microbiological requirement. For some medicinal products this could potentially limit the scope of pharmacovigilance analysis since the information on the intended site “gastric” is excluded.

The study’s findings were used to formalize business rules that detail the process of assigning these attributes and provide guidance on when and how to harmonize medicinal products using global dose form attributes. This led to the harmonization of medicinal products by both removing granular dose form information and applying harmonization rules. Additionally, the aggregated information from this process should improve the efficiency of signal detection in pharmacovigilance and aid in selecting comparable products for cross-border prescriptions and managing drug shortages.

## Conclusion

In summary, global dose form attributes proved to be an innovative and effective tool for describing local and regional dose forms consistently. The flexibility of the global dose form attributes was beneficial in a global setting, as it allowed unrestricted combinations of basic dose form, intended site, administration method, and release characteristics. By including the mapping of local dose forms to global dose form attributes, the assignment process was improved.

One challenge during the study was to identify the correct information associated with each medicinal product. Therefore, there is a need for a secure and robust process of information retrieval from stakeholders such as national regulatory authorities and pharmaceutical companies. To enable this, a sustainable process, including tools for information exchange and collecting points-of-contact, needs to be formalized.

## Future Perspective

To further strengthen the implementation of global dose form attributes, it is essential to broaden the scope of investigated products, dose form terminologies, regions and countries. This will require addressing challenges related to translation from local languages and handling non-Latin alphabets. Additionally, tools for quality control and change management processes, to enable implementation of new or altered EDQM Standard Terms, need to be further developed to maintain the accuracy of the assigned global dos form attributes.

The study highlighted challenges in assigning global dose form attributes due to incomplete or inconsistent information in SmPCs and product labels. Furthermore, the information on release characteristics spread across various sections of the documentation which increased the need for manual review. This underscores the importance of standardized and structured product information to support global harmonization efforts.

The study suggests that while any organization could assign the dose form attributes, the complexity and detailed nature of the process make alignment between organizations challenging. To ensure global harmonization, a single organization must take the lead in evaluating and assigning the global dose form attributes and their corresponding PhPIDs.

## Data Availability

No datasets were generated or analysed during the current study.
